# Effects of bacteriophage traits on plaque formation

**DOI:** 10.1186/1471-2180-11-181

**Published:** 2011-08-09

**Authors:** Romain Gallet, Sherin Kannoly, Ing-Nang Wang

**Affiliations:** 1 Department of Biological Sciences, University at Albany, 1400 Washington Avenue, Albany, New York 12222, USA; 2 CEFE - UMR 5175, 1919 route de Mende, F-34293 Montpellier cedex 5, France

## Abstract

**Background:**

The appearance of plaques on a bacterial lawn is one of the enduring imageries in modern day biology. The seeming simplicity of a plaque has invited many hypotheses and models in trying to describe and explain the details of its formation. However, until now, there has been no systematic experimental exploration on how different bacteriophage (phage) traits may influence the formation of a plaque. In this study, we constructed a series of isogenic λ phages that differ in their adsorption rate, lysis timing, or morphology so that we can determine the effects if these changes on three plaque properties: size, progeny productivity, and phage concentration within plaques.

**Results:**

We found that the adsorption rate has a diminishing, but negative impact on all three plaque measurements. Interestingly, there exists a concave relationship between the lysis time and plaque size, resulting in an apparent optimal lysis time that maximizes the plaque size. Although suggestive in appearance, we did not detect a significant effect of lysis time on plaque productivity. Nonetheless, the combined effects of plaque size and productivity resulted in an apparent convex relationship between the lysis time and phage concentration within plaques. Lastly, we found that virion morphology also affected plaque size. We compared our results to the available models on plaque size and productivity. For the models in their current forms, a few of them can capture the qualitative aspects of our results, but not consistently in both plaque properties.

**Conclusions:**

By using a collection of isogenic phage strains, we were able to investigate the effects of individual phage traits on plaque size, plaque productivity, and average phage concentration in a plaque while holding all other traits constant. The controlled nature of our study allowed us to test several model predictions on plaque size and plaque productivity. It seems that a more realistic theoretical approach to plaque formation is needed in order to capture the complex interaction between phage and its bacterium host in a spatially restricted environment.

## Background

Ever since the discovery of bacteriophages (phages), the prominent clearings that they produce on bacterial lawns (the lysis plaques) have fascinated countless microbiologists. In fact, the name bacteriophage, literally meaning bacteria eater, was derived at least in part from the phage's ability to form clearings [1] (for English translation see d'Hérelle [2]). Besides a few exceptions, such as the phage T7, for which the plaque continues to increase in size [3,4], most phage plaques, after a period of incubation, assume a certain size and acquire a definitive boundary, either with a fuzzy or clear-cut edge. The ability to form plaques is not restricted to phages only since animal and plant viruses also form plaques and lesions on cell cultures [5], host tissues [6], or leaf surfaces [7].

It is usually assumed that each plaque on plates is initiated by a single virus particle, although not all virus particles in the sample can initiate infections [8] and reference therein]. The typical circular plaque morphology is simply the result of cycles of infection of the embedded host cells by the numerous viral progeny disseminating in all directions from the original focus of infection, reminiscent of the traveling wave of an epidemic [9]. With a standardized condition, the plaque morphology can be quite consistent. Therefore, a change in plaque morphology during study is commonly used as an indication that the viral strain may have sustained a mutation(s) [10]. Furthermore, with proper calibration, the phage plaque size has also been used as a surrogate for the fitness measurement [11] (however, see [12]). Plaque size can also be a good indicator of genetic changes for animal viruses [13-15]. More importantly, investigation of plaque formation in a simplified and controlled laboratory condition of an agar gel should allow us to better understand how phages interact with their bacterial hosts in a more natural and complex biofilm environment [16-18].

**Figure 1:**
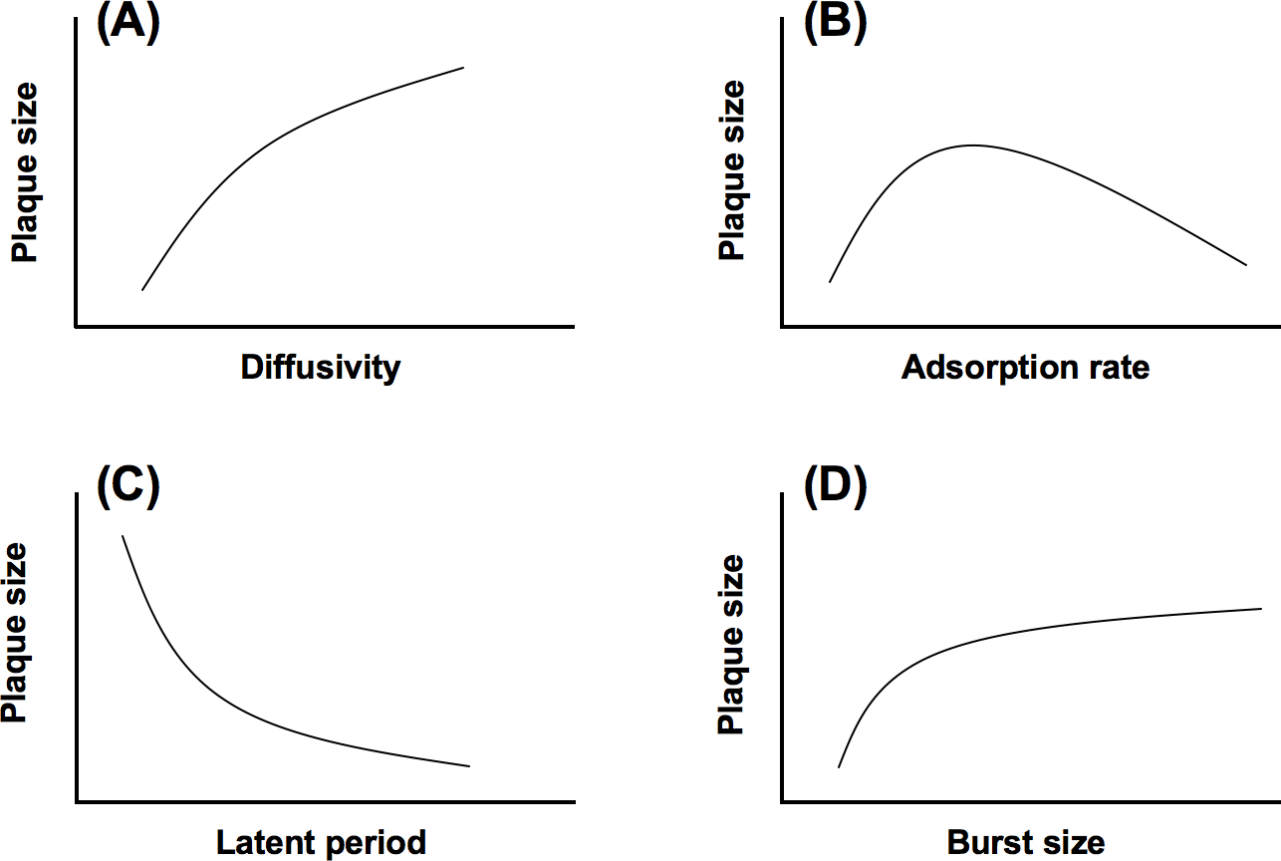
**The expected relationships between plaque size and various phage traits as summarized by Abedon and Yin **[12].

The perceived simplicity of phage plaques has invited several efforts in mathematical modeling. The first of such efforts was pioneered by Koch [19], who approximated the enlargement of a plaque by equating it with the diffusion of phage particles through a fixed host density with either reversible or irreversible adsorption onto the encountered host cells. After a few decades of inactivity by microbiologists, Yin and coworkers [9,20] reinvigorated the effort by incorporating diffusion, adsorption, and production of phage particles into the models. Abedon and coworkers [16,21] have provided an excellent and comprehensive survey of mathematical models on the enlargement of a phage plaque. The commonly considered factors include the virion diffusivity (rate of virion particle diffusion without the presence of the host), various rate constants for phage-bacterium attachment, phage latent period, burst size, and host density. Figure 1 shows the impacts of selected factors on plaque size, as summarized by Abedon and Yin [12]. All else being equal, the phage with a higher diffusivity would have a larger plaque size; specifically the size would be a quadratic function of the diffusivity (Figure 1A). Although the model predictions are not always in total agreement with each other [16], the consensus is that too high or too low an adsorption rate would generally result in a smaller plaque size. That is, there is likely an optimal adsorption rate, leading to a maximal plaque size (Figure 1B). The plaque size is also predicted to be negatively correlated with the latent period (or lysis time), specifically a quadratic function of the latent period (Figure 1C). It is reasoned that the more time the phage progeny spends inside the host, the less time it would be able to diffuse to a new host. It is also intuitively apparent that a larger burst size would result in a larger plaque size. However, simulations [9,20] showed that there is a diminishing impact of burst size on plaque size (Figure 1D).

When compared to studies on plaque size, considerations of plaque productivity, the total number of phage progeny inside a plaque, has received less attention. The most systematic theoretical study was conducted by Abedon and Culler [22]. This was a natural extension of their previous work on phage plaque size [16]. By taking advantage of various mathematical models describing the plaque size enlargement, they were able to derive the relationships between plaque productivity (plaque fecundity, in their terminology) and various phage traits. Not surprisingly, all models predicted that a shorter latent period would result in a lower plaque productivity. However, in some models, the long latent period did not influence the productivity much, thus assuming a plateau-like relationship, while others predicted an optimal latent period, maximizing the plaque productivity [[16]; their Figure 3].

The problem with studies on phage plaque formation is that there are few empirical tests of the various proposed mathematical models [9,19,23]. Most observations are anecdotal, lacking a systematic focus. Typically, only a narrow facet of the model was tested [20]. The main obstacle to conducting experimental tests of these models is that values of various phage traits are not easily changed, unlike in mathematical models and computer simulations. However, the difficulty of experimentally assessing the impacts of phage traits on plaque size and productivity can be overcome by using a series of isogenic phage strains that only differ in one or two traits.

In this study, we constructed and assembled a collection of isogenic λ phage strains that only differed in one, two, or all three phage traits: adsorption rate, lysis time, and morphology. By measuring the plaque sizes with digital image analysis and estimating the plaque productivities of these isogenic phages, we were able to assess the impact of each phage trait while holding other variables constant. We also tested the model predictions using our current results. We found that some of the models were able to capture the empirical results qualitatively but not always quantitatively.

## Results

### Effect of adsorption rate

To assess the impact of adsorption rate on plaque size (surface area of the plaque) and plaque productivity (number of phages per plaque), we constructed eight isogenic strains of phage λ that only differed in their adsorption rate and virion size. This was accomplished by combining four *J *alleles (*J_WT_*, *J_245-2_*, *J_1077-1_*, and *J_1127-1_*) [17,24], which encode the tail fiber proteins (gpJ), and two *stf *alleles (*stf^+ ^*and *stf^-^*), which encode the side-tail fibers (Stf) [17]. Since there is no practical way to determine adsorption rate in the agar gel, we used the rates determined in the liquid culture to serve as surrogates for how these phages would behave in the agar gel. The adsorption rate, as determined here, is a function of phage diffusion coefficient (or diffusivity), which is a function of medium viscosity and phage virion radius [25]. Since all our Stf^+ ^and Stf^- ^phages would have the same shape within the group and experience the same viscosity, therefore we expect the ranking of the adsorption rates within each Stf group to remain the same.

The effect of adsorption rate on plaque size, plaque productivity, and phage concentration in plaques were shown in Table 1. In general, presence of the Stf greatly increases the phage adsorption rate (effect of the Stf status, *p *< 0.0001). But the effects of Stf and J on the adsorption rate are independent from each other (effect of J × Stf status, *p *= 0.81); the ranking of J tail fibers remains the same (gpJ_1077-1 _> gpJ_245-2 _> gpJ_1127-1 _> gpJ_WT_) whether in the presence or absence of the Stf. However, the improvement of the adsorption rate from Stf^- ^to Stf^+ ^is not uniform across all J tail fibers. With gpJ_WT_, which had the lowest adsorption rate, addition of the Stf improved the adsorption rate almost 140-fold; while for gpJ_1077-1_, which had the highest adsorption rate, addition of the Stf only gained about 8-fold improvement.

**Table 1:** Effects of adsorption rate on plaque size, plaque productivity, and phage concentration in plaque.

Relevant phenotype	Adsorption rate ± 95% CI (× 10^-10 ^mL/min)	Plaque size ± 95% CI (mm^2^)	Plaque productivity ± 95% CI (× 10^6 ^phages/plaque)	Phage concentration in plaque ± 95%CI (× 10^8 ^phages/mL)
Stf^+ ^J_WT_	102.60 ± 29.81	1.73 ± 0.17	2.92 ± 1.27	33.10 ± 12.70
Stf^+ ^J_1127-1_	118.10 ± 31.64	1.51 ± 0.19	0.38 ± 0.13	9.20 ± 8.49
Stf^+ ^J_245-2_	128.30 ± 43.57	1.21 ± 0.21	0.40 ± 0.11	6.92 ± 2.43
Stf^+ ^J_1077-1_	139.50 ± 45.96	1.05 ± 0.14	0.19 ± 0.07	3.64 ± 1.42
Stf^- ^J_WT_	0.74 ± 0.72	3.36 ± 0.61	84.20 ± 27.00	486.00 ± 91.00
Stf^- ^J_1127-1_	5.09 ± 2.52	2.14 ± 0.19	3.64 ± 0.62	34.30 ± 6.27
Stf^- ^J_245-2_	10.22 ± 5.26	2.55 ± 0.42	5.53 ± 1.89	43.60 ± 12.70
Stf^- ^J_1077-1_	18.49 ± 8.21	2.02 ± 0.33	3.61 ± 4.03	32.50 ± 31.10

**Figure 2:**
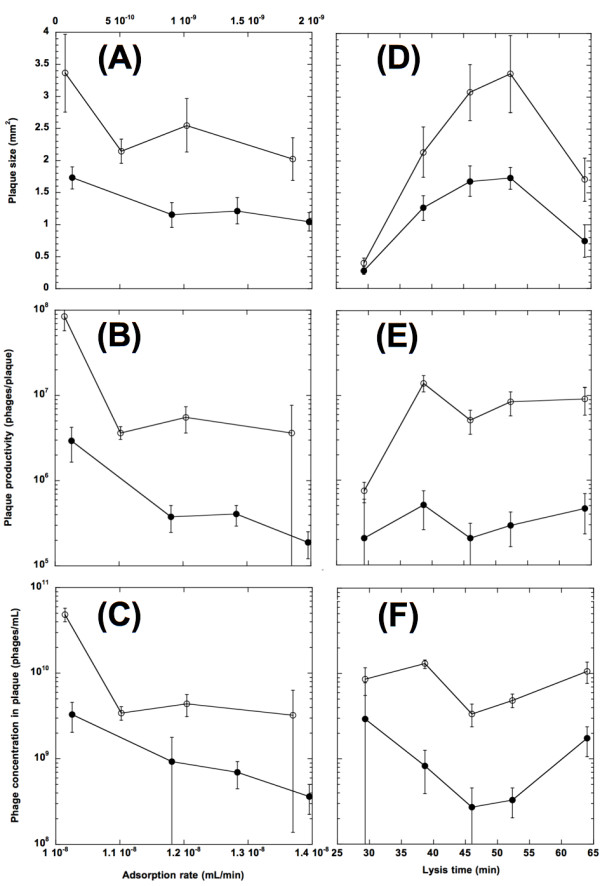
**Effects of phage adsorption rate and lysis time on plaque size, productivity, and concentration in plaques**. Plaque size (A and D), plaque productivity (B and E), and phage concentration within plaques (C and F) are plotted against either the adsorption rate (A - C; top *x*-axis for the Stf^- ^phages, bottom *x*-axis the Stf^+ ^phages) or the lysis time (D - F). In all cases, Stf^+ ^phages (filled circles) and Stf^- ^phages (open circles) are plotted separately. Error bars showed the 95% confidence intervals.

As shown in Figure 2A and 2B, both the plaque sizes (Stf^+^: *F*[1,34] = 29.77, *p *< 0.0001; Stf^-^: *F*[1,32] = 12.91, *p *= 0.0011) and plaque productivity (Stf^+^: *F*[1,34] = 33.99, *p *< 0.0001; Stf^-^: *F*[1,32] = 19.87, *p *< 0.0001) were negatively impacted by the adsorption rate. As reported previously [17], when compared to the low-adsorption phages, the high-adsorption phages produced smaller plaques and fewer progeny per plaque. It is also interesting to note that, when compared to their Stf^- ^counterparts, the presence of the side-tail fibers, which greatly increases the adsorption rate (see above), contributed a relatively consistent two-fold reduction in plaque size and a range from 10- to 29-fold reduction in plaque productivity across all *J *alleles.

It is possible that the phage concentration inside the plaque is the same across all the phage strains; and therefore that the reason why the high-adsorption phages produced fewer phages per plaque is because they made smaller plaques. We tested this possibility by estimating the phage concentrations inside the plaques. Since we did not directly measure the volume of each plaque, we made the following assumptions: the shape of the plaque would be cylindrical with a height of 0.5 mm if its average radius is equal or larger than 0.5 mm, otherwise the shape would be semi-spherical. The rationale for the assumption is based on the fact that the Petri dish used for phage plating has an inner diameter of ~8.7 cm and the volume of the top agar is ~3 mL. That is, the thickness of the top agar layer would be about 0.5 mm in height. By further assuming that all seedings of the originally infected host cells are taking place on top of the top agar layer, we can calculate the average plaque volume for each phage strain. In this particular case, all phage strains have an average plaque radius larger than 0.5 mm. As shown in Figure 2C, our result showed that the higher the adsorption rate then the lower the phage concentration within plaques (Stf^+^: *F*[1,34] = 33.74, *p *< 0.0001; Stf^-^: *F*[1,32]= 23.78, *p *< 0.0001).

Inspection of Figures 2A-2C also reveals a pattern of adsorption rate having a diminishing impact on all three plaque properties. Omission of either gpJ_WT _strain (the phage with the lowest adsorption rate in either the Stf^+ ^or Stf^- ^background) from analyses however showed that there is no significant effect of the adsorption rate on plaque properties, except for the productivity of the Stf^+ ^phages (analyses not shown). This observation suggests that once the adsorption rate exceeds a certain value, any further increase would not make much difference in plaque formation.

### Effect of lysis timing

Lysis time (or latent period) determines the duration of the intracellular phase of phage production before cell lysis. Generally, there is a positive linear relationship between the lysis time and burst size [26]. Therefore, the impact of lysis time on plaque size, plaque productivity, and phage concentration within plaques would also be mediated through its accompanying effect on burst size. Notwithstanding this complication, to elucidate the interaction between adsorption rate and lysis time, and their joined effects on phage plaque size and plaque productivity, we constructed isogenic λ strains that differed in their adsorption rates (through the presence or absence of the Stf, but also the virion size as well, see below) and lysis times (due to different holin gene *S *alleles). This collection of isogenic strains used for this purpose has been described elsewhere [27].

The effects of lysis timing on plaque size, plaque productivity, and phage concentration in plaques were shown in Table 2. As shown in Figure 2D, the long and short lysis-time phages made smaller plaques than the medium-lysis time phages for both the Stf^+ ^and Stf^- ^phages. In fact, there is an apparent optimal lysis time between 45 to 50 min that maximizes the plaque size for both the Stf^+ ^and the Stf^- ^phages (both quadratic fits; Stf^+^: *F*[2,41] = 114.78, *p *< 0.0001; Stf^-^: *F*[2,40] = 90.27, *p *< 0.0001). Once again, the Stf^+ ^phages have a consistently smaller plaque size when compared to their Stf^- ^counterparts. As in the case of the *J *alleles described above, the presence of the Stf also contributed to approximately a two-fold reduction in plaque size (results not shown), except in the case of the shortest lysis time variant, for which the plaque sizes are similar to each other, though still statistically different (*F*[1,15] = 7.70, *p *= 0.014). Unlike in the case of plaque size, for both the Stf^+ ^and Stf^- ^phages, the lysis time makes no apparent difference in plaque productivity (Stf^+^: *F*[1,42] = 0.66, *p *= 0.421; Stf^-^: F[1,41] = 2.66, *p *= 0.110) (Figure 2E).

**Table 2:** Effects of lysis timing on plaque size, plaque productivity, and phage concentration in plaque.

Relevant phenotype	Lysis time^1 ^± 95% CI (min)	Plaque size ± 95% CI (mm^2^)	Plaque productivity ± 95% CI (× 10^6 ^phages/plaque)	Phage concentration in plaque^2 ^± 95%CI (× 10^10 ^phages/mL)
Stf^+ ^S_M1L/C51S/S76C_	29.3 ± 1.47	0.28 ± 0.06	2.08 ± 3.90	2.94 ± 4.84
Stf^+ ^S_M1L/C51S_	38.7 ± 1.47	1.27 ± 0.19	5.09 ± 2.48	0.82 ± 0.43
Stf^+ ^S_M1L_	46.0 ± 0.00	1.68 ± 0.24	2.07 ± 1.06	0.27 ± 0.19
Stf^+ ^S_WT_	52.3 ± 1.27	1.73 ± 0.17	2.92 ± 1.27	0.33 ± 0.13
Stf^+ ^S_S68C_	64.0 ± 0.00	0.74 ± 0.25	4.61 ± 2.28	1.73 ± 0.66
Stf^- ^S_M1L/C51S/S76C_	29.3 ± 1.47	0.40 ± 0.08	7.47 ± 2.04	8.55 ± 3.07
Stf^- ^S_M1L/C51S_	38.7 ± 1.47	2.14 ± 0.39	140.00 ± 30.70	13.00 ± 1.50
Stf^- ^S_M1L_	46.0 ± 0.00	3.07 ± 0.44	50.70 ± 15.70	3.38 ± 1.00
Stf^- ^S_WT_	52.3 ± 1.27	3.36 ± 0.61	84.20 ± 27.00	4.86 ± 0.91
Stf^- ^S_S68C_	64.0 ± 0.00	1.71 ± 0.33	91.10 ± 32.10	10.60 ± 2.94

^1 ^The lysis times and 95% confidence intervals were reprinted from [27], Table 2.

^2 ^Note the multiplier for phage concentration in plaque is 100-fold higher than that used in Table 2.

Not surprisingly, the estimated plaque volumes are quite different among different lysis-time variants (data not shown). In this case, all lysis-time variants were assumed to have a cylindrical shape, except for the shortest lysis-time strains, which were assumed to be in the semi-spherical shape (see above for rationale). Since the plaque productivities are similar among the lysis time variants, while the plaque volumes are mainly correlated with the plaque size, it is not surprising to observe that the relationship between the lysis time and phage concentration within plaques for both the Stf^+ ^and the Stf^- ^phages is apparently convex (Figure 2F). However, quadratic fits show a barely significant effect of lysis time on phage concentration within plaques for the Stf^+ ^phages (*F*[2,41] = 2.80, *p *= 0.073), but a significant effect for the Stf^- ^phages (F[2,38] = 6.14, *p *= 0.005). Both fits showed that the minimum phage concentration within plaques is located around 45 to 50 min.

Our results show that the lysis time has a concave relationship with plaque size for both the Stf^+ ^and the Stf^- ^phages (Figure 2D). But no apparent significant impact on plaque productivity was found (Figure 2E). Also, there seemed to be a convex relationship between the lysis time and the phage concentration within plaques (Figure 2F). Apparently, and unlike the adsorption rate, lysis time has a much more complex influence on various plaque properties. However, this may not be a surprising outcome, for lysis time is positively correlated with the burst size [26]. Thus variation in lysis time would inevitably affect the burst size as well.

### Effect of phage morphology

Besides providing a high adsorption rate, the presence of the Stf would presumably reduce the phage's ability to diffuse freely through the top agar layer. This is due to the extra side tail fibers extending from the virion, potentially increasing the hydrodynamic drag of the phage particle. However, the effect of phage morphology on plaque size cannot be tested simply by comparing between phages with and without the Stf. This is because the Stf has the dual effect of increasing the adsorption rate and reducing the phage diffusion at the same time. To separate the effect of adsorption rate from morphology, we took advantage of the fact that the host surface receptor for the Stf is the OmpC protein (data not shown). When using an *ΔompC*::*kan *strain, the Stf^+ ^and the Stf^- ^phages had indistinguishable adsorption rates when determined in liquid culture (data not shown). It was reasoned that by using an *ΔompC*::*kan *strain, the difference in plaque formation between the Stf^+ ^and Stf^- ^strain would be due solely to the phage morphology.

To test the above hypothesis, one strain of the Stf^+ ^and the Stf^- ^phages (both carrying the wt *J *and *S *alleles) were used. We expect that

(*i*) For the Stf^+ ^phage, plaques on the wild-type (wt) host should be smaller than those on the ΔOmpC host. This is because when on the wt host the Stf^+ ^phage would have a higher adsorption rate. But for the Stf^- ^phage, plaques should have the same size on both the wt and the ΔOmpC host. This is because the Stf^- ^phage would have the same adsorption rate and virion size on either host.

(*ii*) When plated on the wt host, the Stf^+ ^phage should have smaller plaques than those of the Stf^- ^phage. This is because the Stf^+ ^phage would have a higher adsorption rate and a larger virion size, both contributing to the making of a smaller plaque. On the other hand, when plated on the ΔOmpC host, the Stf^+ ^phage should have smaller plaques than those of the Stf^- ^phage. This is because the Stf^+ ^phage would have a larger virion size, due to the presence of the Stf.

(*iii*) Furthermore, when plated on the ΔOmpC host, the size difference between the Stf^+ ^and the Stf^- ^phages should be smaller than that when on the wt host. Again, when on the ΔOmpC host, the difference should simply be due to the virion size only, while when on the wt host, both the adsorption rate and the virion size would contribute to the difference.

**Figure 3:**
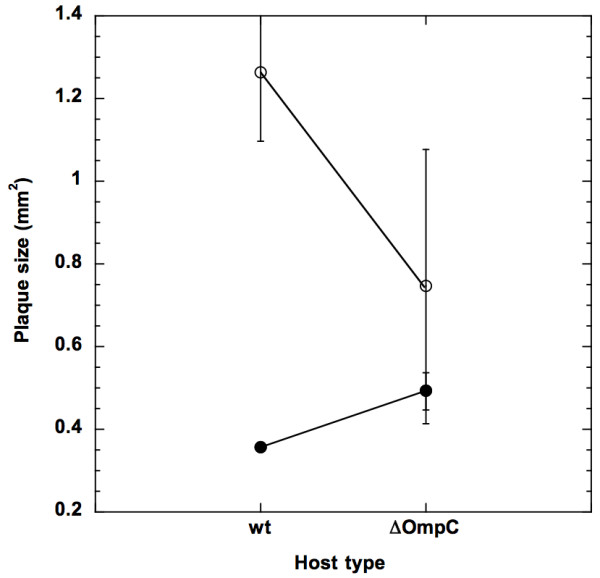
**Effecs of host type and Stf on plaque size**. Plaque sizes were determined for the Stf^+ ^(filled circles) and Stf^- ^(open circles) by plating on either the witld type (wt) or the ΔompC (ΔOmpC) *E. coli *cells. Error bars showed the 95% confidence intervals. Horizontal solid lines intend to show the size differences from the same phages when plated on different host.

Figure 3 summarizes our results. For expectation (*i*), we observed that the Stf^+ ^phage made a smaller plaque when plated on the wt host, as opposed to the ΔOmpC host (0.36 vs. 0.49 mm^2^; *F*[1,8] = 72.25, *p *< 0.0001). However, quite unexpectedly, the Stf^- ^phage made a smaller plaque when plated on the ΔOmpC host, as opposed to the wt host (0.75 vs. 1.26 mm^2^; *F*[1,8] = 14.98, *p *= 0.005). For expectation (*ii*), we observed that, when plated on the wt host, the Stf^+ ^phage made a smaller plaque when compared to the Stf^- ^phage (0.36 vs. 1.26 mm^2^; *F*[1,8] = 232.07, *p *< 0.0001). However, when plated on the ΔOmpC host, we only observed a borderline significant level of plaque size difference between the Stf^+ ^and Stf^- ^phages (0.49 vs. 0.75 mm^2^; *F*[1,8] = 4.45, *p *= 0.068; however, the non-parametric Wilcoxon/Kruskal-Wallis test showed a significant difference, *z *= -2.01, *p *= 0.034 for the one-way test). For expectation (*iii*), we observed that the plaque size difference between the Stf^+ ^and Stf^- ^phages is significantly larger when plated on the wt host (3.5-fold, with 95% confidence interval of 3.15 - 3.92-fold vs. 1.5-fold, with 95% confidence interval of 0.95 - 2.10-fold), indicating that a larger virion, as a result of having extra appendages, would retard virion diffusion through the top agar layer, thus reducing the plaque size.

### Testing model predictions on phage plaque size and productivity

Abedon and Culler [16,22] reviewed seven mathematical models on phage plaque enlargement, as listed in the Appendix. Unfortunately, these models cannot be tested directly with our current data. This is because all the models required the parameter of virion diffusivity, a quantity we did not measure in this study. However, by taking advantage of our identical experimental condition and various isogenic phage strains that only differed in selected traits, we can nevertheless test the relative impacts of various phage traits on plaque formation and progeny production in the plaques. We reasoned that the plaque radius *r *or plaque productivity *p *can be expressed as functions of phage traits so that *r *= *f*(*a, L*, *D*) and *p *= *g*(*a*, *L*, *D*), where *a *is the adsorption rate, *L *the lysis time, and *D *the phage diffusivity. For isogenic phage strains that only differ in adsorption rates, the expected ratios of *r_1_*/*r_2 _*and *p_1_*/*p_2 _*can be simplified as *r_1_*/*r_2 _*= *f*(*a_1_*, *L*, *D*)/*f*(*a_2_*, *L*, *D*) = *f*(*a_1_*)/*f*(*a_2_*) and *p_1_*/*p_2 _*= *g*(*a_1_*, *L*, *D*)/*g*(*a_2_*, *L*, *D*) = *g*(*a_1_*)/*g*(*a_2_*). Also, for isogenic strains that only differed in lysis times (with the concomitant differences in burst size), the expected ratios of *r_1_*/*r_2 _*and *p_1_*/*p_2 _*can be simplified as *r_1_*/*r_2 _*= *f*(*a*, *L_1_*, *D*)/*f*(*a*, *L_2_*, *D*) = *f*(*L_1_*)/*f*(*L_2_*) and *p_1_*/*p_2 _*= *g*(*a*, *L_1_*, *D*)/*g*(*a*, *L_2_*, *D*) = *g*(*L_1_*)/*g*(*L_2_*). The resulting model predictions can then be compared against our observed data. The exact model predictions for both the plaque size and plaque productivity are listed in the Additional file 1.

Since virion morphology is likely to impact plaque formation (see above), we only conducted comparisons within each morphology group, using the wt λ*stf^+ ^*or the wt λ*stf^- ^*as the denominators for the ratio comparisons.

**Figure 4:**
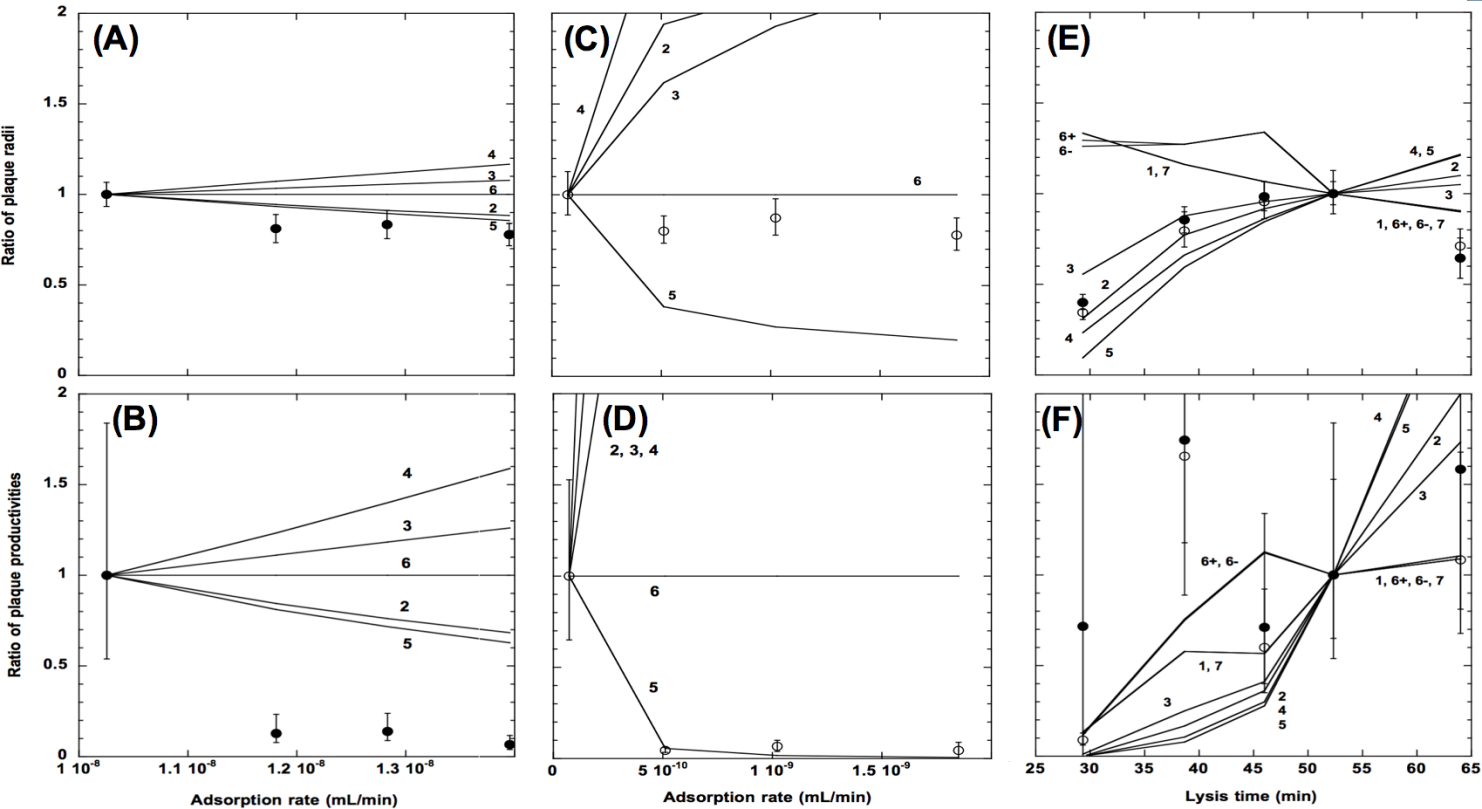
**Observed and expected ratios of plaque radius and plaque productivity**. Ratios of plaque radii (A, C, and E) and plaque productivity (B, D, and F) are plotted against adsorption rate (A - E) or lysis time (E and F). Solid lines and numbers showed the model predictions from equations listed in Table A.2. Filled circles denote observed ratios from the Stf^+ ^phages and open circles the Stf^- ^phages. Plus and minus signs next to the numbers indicate Stf^+ ^phages and Stf^- ^phages, respectively. All values are compared against those of the wild type λ, with or without the Stf. Error bars denote the 95% confidence intervals of the observed ratios (see Methods).

For both the Stf^+ ^(Figure 4A) and Stf^- ^(Figure 4C) phages, the observed ratios of plaque radii--obtained as the ratios of the square roots of the determined plaque surface areas--did not vary greatly with the adsorption rate. However, except for Eqn. 5, and Eqn. 2 (see Appendix) when in high adsorption rate, both of which predicted a declining ratio as adsorption rates increased (Figure 4A). However, all other models listed in the Appendix failed to predict observed ratios of plaque radii. The failure is especially prominent when the adsorption rate is low, *i.e*. for the Stf^- ^phages (Figure 4C).

For isogenic phage strains that differed in their lysis times (and burst sizes), the ratios of plaque radii also showed the same peaked pattern (Figure 4E) shown in Figure 2D. Interestingly, both the Stf^+ ^and Stf^- ^phages showed the same ratios of plaque radii, even though the Stf^+ ^phages generally have significantly smaller plaque sizes (Figure 2A). Furthermore, unlike the above result, Eqn. 3 seemed to perform reasonably well in predicting ratios of plaque radii, at least when the lysis time is shorter than 52.3 min. All the models predicted a larger ratio than observed when the lysis time is longer than 52.3 min.

As the adsorption rate increases, the observed ratios of plaque productivity declined to a similar degree for both the Stf^+ ^(Figure 4B) and Stf^- ^(Figure 4D) phages. However, except for Eqn. 5, which performed superbly when the adsorption rate is low (Figure 4D), none of the other models can reasonably predict the observed ratios. As before, the failure is more prominent when the adsorption rate is low.

For the strains with different lysis times, both the Stf^+ ^and Stf^- ^phages showed an almost identically complex pattern, except when the lysis time is very long or very short (Figure 4F). However, due to uncertainty in our productivity data, the performance of each model is harder to assess. However, even with such a high uncertainty, none of the models can predict the plaque productivity within the entire range of lysis time used in our study. This is especially true when the lysis time is ~39 min.

## Discussion

The appearance of a plaque is the oldest, but also the most useful and direct way of confirming the presence of a phage. Even with the advent of modern technologies, such as real-time quantitative PCR and fluorescence-labeling, the simplicity of plaque counting is still the easiest and the most commonly used method for quantifying the number of infectious phages in a sample [28,29]. Even in the earliest days, researchers have been divining the various idiosyncratic traits of a phage through the size and shape of the plaque it makes [30].

Except for plaques made by phages like T7, most plaques have a definitive size after overnight incubation. One of the most important changes during this typical incubation period is the switch of host physiology from the initial exponential growth to the eventual stationary stagnation. With few exceptions [3,4,31], most phages cannot sustain productive infections when infecting stationary phase cells. Consequently, the plaque size would be limited by the amount of time available for productive infections. The length of productive time can be manipulated by either the initial host density or host physiology (*e.g*., growth rate). For example, in the case of phage ϕ6, the phage made a larger plaque when plated with a lower initial host density [19,32]. In the most extreme case, addition of sub-lethal amount of antibiotics and/or glycerol in the agar plate, presumably changing the host physiology, greatly improved the appearance of the plaque, transforming it from small and turbid to large and clear [33]. In our study, however, all the plating conditions were kept constant (except when determining the impact of phage morphology on plaque size, in which we used different host strains), therefore, the differences in plaque size and productivity would simply be due to the differences in phage traits, rather than the amount of time available for productive infection.

The life cycle of a phage in an agar plate can be divided into two parts: the extracellular phase for virion diffusion/adsorption and the intracellular phase for progeny production. All else being equal, more time for the extracellular phase would allow the virion to diffuse farther. On the other hand, more time for the intracellular phase would produce more progeny that could be diffused. From this point-of-view, it can be argued that the problems of plaque size and plaque productivity can be seen as a problem of how to optimally allocate the limited time between the extra- and intra-cellular phases. It is possible that the optimal time allocation for maximum plaque size may not be the same for maximum plaque productivity [22].

### Effects of adsorption rate

At present, our results generally show a diminishing negative impact of the adsorption rate. These results do not entirely fit the expectation of the consensus [12], which predicts an optimal adsorption rate that maximizes the plaque size (Figure 1B). One possible explanation for the discrepancy is because our phage collection has a narrower range of adsorption rates than those used in the models. Consequently, the observed diminishing negative relationship could simply be a reflection of the fact that all our phages have medium to high adsorption rates when compared to the model simulations. Though whether this is the case remains to be seen, it should be pointed out that it makes an intuitive sense that a lower adsorption rate, at some point, should result in a smaller plaque size. After all, for a phage with a very low adsorption rate, it would spend proportionally more time in the extracellular phase diffusing before it initiates an actual infection. By the time the phage clears enough host cells to reveal a visible plaque, the host physiology may have already switched to the unproductive phase. That is, for a phage with a very low adsorption rate, the plaque would be small, and possibly blurry, due to host over-growth (Abedon, per. comm.; [19] for smaller plaques due to lowered adsorption rate via withholding cofactor; [34] and [35] for low adsorption rate and turbid plaques in *ht *mutants).

Because the ratio tests of each model showed that none of these models could consistently reproduce the observed ratios of plaque radius and plaque productivity (Figure 4), it suggests that other factors may also be important in the formation of a plaque. For example, for a high-adsorption phage, the time spent in the extracellular phase would be shorter when compared to a low-adsorption one. That is, there would be less time for a high-adsorption phage to diffuse too far away from where it was released before it encounters another host cell. Consequently, on average, a higher proportion of the released progeny would be adsorbed onto the cells that are in their immediate vicinities. There are several consequences from such a scenario: (*i*) One likely consequence of the high adsorption rate in a spatially restricted environment is that many of the host cells nearby would be multiply infected. Multiple infection would potentially shorten the lysis time (the latent period) by producing more holin proteins inside the cell [36]. On the other hand, it may also increase the burst size per infected cell because more genomes would contribute to the synthesis of virion components. For example, infection of phage λ to *E coli *strains expressing λ's morphogenetic genes *B*, *D*, or *W *would increase 20 to 40% of the normal burst size (Shao & Wang, unpublished data). But the progeny produced per infected phage would likely be lower than when the host is singly infected (for phage ϕ6, P. Turner, per. comm.). It is possible that in our case the balance is tilting toward an overall reduction of progeny output per unit of time spent inside the host cell during multiple infections. (*ii*) Besides the elevated chance of multiple infection, a shorter travel distance would also likely lead to the phenomenon of "self shading," [37,38] where a cell infected by a high-adsorption phage is likely to be surrounded by host cells also infected with the high-adsorption phage. Consequently, for a given number of the progeny, less distance is traveled (diffused), leading to a smaller plaque size and less host cells are encountered, leading to a smaller productivity. (*iii*) One consequence of the localized infection is the concentration of localized cell debris, which has been theorized to affect host and phage dynamics [39,40]. Our preliminary result showed that the infectivity of phage λ can be inhibited by cell debris (unpublished data). Therefore, not only a high-adsorption phage is likely to adsorb onto a host cell, it is also likely to encounter cell debris scattered around in its vicinity, thus reducing the overall progeny production through dead-end infection. It would be interesting to see if incorporation of these factors would alter the predicted effect of adsorption rate much.

### Effects of lysis time

One of the most interesting findings in this study is the concave relationship between the lysis time and the plaque size (Figure 2D), with the long- and the short-lysis time phages making smaller plaques than the medium lysis time phages. While this pattern mirrored the relationship between the lysis time and phage fitness (growth rate) [26,27], nevertheless, there is one important exception: namely, in the case of the phage fitness, the optimal lysis time depends on the adsorption rate while, in the case of the plaque size, the optimal lysis time is independent of the adsorption rate.

It is understandable why a phage with a longer lysis time would make a smaller plaque. After all, more time spent in producing progeny inside the host means that less time is available for diffusing among the host cells. However, at first glance, it is not immediately clear why a shorter lysis time would also result in a smaller plaque. The most likely explanation is that a shorter lysis time is usually correlated with a smaller burst size [26,41-43]. A smaller burst size means that less progeny are available for diffusion, hence a smaller plaque. The bust size of the shortest lysis time strain in our study is ~10 phages/cell [26,27]. This extremely low burst size, as a result of the short lysis time, has two consequences. Firstly, the plaque size becomes relatively indifferent to the adsorption rate. A closer inspection of Figure 2D revealed that the shortest lysis time phage, whether carrying the Stf or not, made much more similarly sized plaques when compared to other lysis time variants (see Results). This indicates that, when under the condition of very short lysis time (thus a very small burst size), the plaque size is mainly determined by the number of progeny available for diffusion, rather than the retardation from the adsorption rate. Secondly, based on our anecdotal observation, a high proportion of the plaques made by the shortest lysis time phages are quite irregular in shape, many times looking like a budding potato instead of the usual circular shape. This, again, is consistent with the hypothesis that not enough of the progeny are available for diffusion to all directions. (On the other hand, it is also possible that the irregular shape is a result of phage evolution within a plaque [4,44]. However, the plaque morphology of our shortest lysis time variant is much more dramatic than simply a general circular shape with slight irregular edges.) Therefore, even though both the long and the short lysis time phages would make small plaques, but the reasons are different. For the short lysis time phages, the main determinant of the plaque size is the number of available progeny for diffusion, while for the long lysis time phages, it is the available time for diffusion that is limiting. The maximum plaque size is thus a compromise between prolonging the lysis time to make enough progeny for diffusion and reducing the lysis time to allow enough extracellular time for virion diffusion.

Even though we do not have an *a priori *expectation on what the relationship between lysis time and plaque productivity would be (because all the models treat the lysis time and burst size as two independent variables, while in our experimental system these two are positively correlated), it is still somewhat surprising that we did not observe any significant effect of lysis time for both the Stf^+ ^and the Stf^- ^phages (Figure 2E). One possible *ad hoc *explanation is that, per unit of time, a short-lysis time variant would experience more cycles of infection but with less progeny participating in each cycle (because of the low burst size), while for a long-lysis time variant the opposite is true. In the end, the productivities remained constant. As a consequence, we observed the convex relationship between the lysis time and phage concentration within plaques. However, another possibility, suggested by closer inspection of Figure 2E, is that the relationship between lysis time and plaque productivity is a complex one, which would require nonlinear fits of *a priori *models to be unmasked. It would be extremely informative if an analogous set of isogenic phages, possibly with a different range of lysis time and burst size, could be constructed to test against our finding that the plaque productivity is in general indifferent to lysis time variation.

### Effects of virion morphology

We were somewhat surprised to find only a borderline significant effect of virion morphology on plaque size. This is because, all else being equal, we expect that a larger phage particle (the Stf^+ ^phage) would diffuse more slowly than a smaller one (the Stf^- ^phage), thus resulting in a smaller plaque. Since we have not measured the diffusivity of either phage in the top agar gel, we do not know how different these two values would be. One possible explanation for the lack of strong morphology effect could be that the size and shape of the Stf^+ ^and the Stf^- ^phages are quite similar to each other. Thus they would have a similar diffusivity, consequently a similar plaque size. This explanation implies that the different plaque sizes when plated on the wt host is mainly due to the difference in adsorption rate between the Stf^+ ^and Stf^- ^phages, not the virion size.

On the other hand, the dramatic size difference for the Stf^- ^phage when plated on the wt and the ΔOmpC hosts (Figure 3) is unexpected. It is possible that the in-frame insertion of the *kan *marker into the *ompC *gene [45] may have disturbed the cell physiology somehow, possibly by interfering with pH and osmolarity regulation, both of which have been implicated as part of OmpC's functions [46,47]. Reduced expression of OmpC has also been linked to a lower activity of the σ^E^, a sigma factor involved in *E. coli*'s stress response [48]. Consequently, there is a general depressive effect on plaque size when plated on this particular ΔOmpC host.

It seems that a more conclusive test of whether phage λ's Stf could significantly impact plaque size or not would be to use a different OmpC mutant that is physiologically equivalent to the wt strain, which can be judged by the similarity of plaque sizes when plated with the Stf^- ^phage. Such a mutation could theoretically be obtained by selecting for *E. coli *mutant that is resistant to the distal part of phage T4's long tail fiber, gp37, which has been shown to be homologous to λ's Stf [49].

### Model performance

Generally, every model reviewed by Abedon and Culler [16,22] failed one way or another to predict plaque size or plaque productivity with our ratio comparisons. The failure could ostensibly be due to assumptions we made in constructing these tests. For example, while models proposed by Yin and McCaskill [20] and Ortega-Cejas *et al*. [23] all took consideration of host density in the bacterial lawn, the density is assumed to be constant. We used the empirically determined ~8.5 × 10^8 ^cells/mL in cases where the host density is required for prediction (*e.g*., eqns 2 and 6 in the Appendix). It is possible that the growth of a bacterial lawn during the incubation period would result in model failure. However, substituting the empirical cell density to a value of 10-fold lower or higher did not improve model performance (data not shown). In fact, several models did not even have the final host density as a variable in ratio comparisons (see the additional file 1).

Another source that may contribute to model failure is the adsorption rates used. Ideally we would want to estimate adsorption rate in the top agar, a technically challenging endeavor that may not be easily achieved. However, we expect that the impact of top agar on the actual adsorption rates should be similar across our isogenic Stf^+ ^and Stf^- ^strains. By using the first-order rate equations to describe the reactions of  (where *B*, *P*, *BP*, and BP* are bacteria, free phage, transient, and stable phage-bacterium complexes, respectively), Moldovan *et al*. [50] estimated the adsorption (*k*), desorption (*k'*), and irreversible-binding rates for phage λ to be at the orders of 10^-11 ^(mL/s), 10^-3 ^(1/s), and 10^-3 ^(1/s), respectively (their Table 1). Therefore, for phage λ, it is the initial recognition between the phage tail fiber and bacterial receptor that is the "rate-limiting" step in phage adsorption. That is, the different adsorption rates among our isogenic λ strains are likely due to differences in *k*, rather than *k' *or *k''*. It is unlikely that the presence of agar in the immediate vicinity of a phage virion and a bacterium would drastically alter the recognition process. Even though agar is much more viscous than the liquid medium, the phage diffusivity in agar should be impacted to the same degree across all our Stf^+ ^or Stf^- ^phages, as described by the Stokes-Einstein equation [50-52], which stated that the solvent (agar) viscosity and the solute diffusion coefficient (phage diffusivity) are inversely related to each other. Taken together, it seems probable that even if the adsorption rate estimated in agar is different from the one estimated in liquid culture, the difference may not be too large.

In our ratio comparisons, we used the endpoint plaque size for our test, rather than the velocity of plaque wavefront, which is what has actually been modeled. It is not clear how this discrepancy may contribute to model failure. But it is to be noted that, except in few cases like phage T7, the velocity of plaque wavefront may not be as easily determined as the endpoint plaque size (but see [53]).

Many of the models are simplified versions of a much complex general model, therefore, their predictions are only valid under restricted conditions. The failure of model predictions may simply reflect the fact that our experimental conditions violated the model assumptions. However, the almost universal failure of all models suggests that it may not be simply the result of assumption violations.

### Implications for phage ecology and evolution

The plaque size, productivity, and concentration are all aftereffects of the combined action of various phage traits. However, except in the case of artificial selection for, say, large plaque size for ease of manipulations [54], it is not clear how natural selection would act on these aftereffects so that various phage traits could be selected as a result. One possible selection scenario is the periodic destruction of biofilm habitat and its concomitant dispersion of the phage inhabitants. The experimental equivalent of this scenario is the homogenization of the top agar gel containing plaques and the extraction of the total phages for subsequent plating. Under such a scenario, the plaque productivity would be the target of selection. That is, the high-production strain would out-compete the low production ones. Since adsorption rate is negatively associated with the plaque productivity, evolution of the adsorption rate would then be toward the lower end of the spectrum. It is to be noted that this scenario provides another advantage of being a low-adsorption phage in the biofilm environment that is different from what has been shown previously. In the prior case, the advantage of a low adsorption rate is manifested through its increased ability to diffuse out of the current plaque, thus greatly increasing the proportion of the individuals that can successfully emigrate out the current location [17]. Any selection scenarios that would target plaque size or phage concentration in the plaques should have a similar effect on the evolutionary trajectory of the adsorption rate.

This simple rule-of-thumb for the evolution of phage traits in a spatially restricted environment may not be applied to the lysis time. This is because plaque productivity seems to be indifferent to lysis time variation, at least over the range covered in our study. This observation would imply that selection for plaque productivity in such an environment would not result in the evolution of lysis time. This is in contrast to our previous study which showed that lysis time is important in phage production when in liquid culture [26,27].

## Conclusions

Our experimental study examined the effects of phage traits on various plaque properties. We showed that adsorption rate negatively impacts plaque size, plaque productivity, and phage concentration in plaques. On the other hand, the plaque size is at its maximum when the lysis time is intermediate in length. But differences in lysis time did not significantly influence plaque productivity. Moreover, the phage with an expected larger virion size showed a smaller plaque size. However, available mathematical models on plaque size and plaque productivity, in their current forms, did not consistently capture the general trends revealed in our study, suggesting that more works are needed to incorporate realism into model description of plaque formation.

## Methods

### Bacterial and phage strains, plasmids, and primers

Bacterial and phage strains used in this study are listed in Table 3. Plasmids and primers are listed in the Additional file 2. Bacterial cultures were grown in LB medium with antibiotics when appropriate.

**Table 3:** List of bacterial and phage strains used in this study.

Name	Relevant genotype	References
XL1 Blue	*lacZΔM15*	Stratagene
SYP052	MC**4100(λ*cI857 S_wt _Δ*(*J-orf401*):**:*cam*)	[27]
IN714	JW2203 (CGSC#9781), *ΔompC768::kan *	[45]
SYP124	MG1655 with *lacZΔM15*	Shao (unpublished data)
IN731	SYP124 with *ΔompC768::kan lcaZΔM15*	This study
SYP045	MC4100(λ*cI857 Swt R::LacZα^+ ^J_WT _stf^-^*)	[27]
SYP046	MC4100(λ*cI857 Swt R::LacZα^+ ^J_WT _stf^+^*)	[27]
SYP085	MC4100(λ*cI857 S_S68C _R::LacZα^+ ^J_WT _stf ^-^*)	[27]
SYP093	MC4100(λ*cI857 S_S68C _R::LacZα^+ ^J_WT _stf^+^*)	[27]
SYP086	MC4100(λ*cI857 S_M1L _R::LacZα^+ ^J_WT _stf ^-^*)	[27]
SYP094	MC4100(λ*cI857 S_M1L _R::LacZα^+ ^J_WT _stf^+^*)	[27]
SYP087	MC4100(λ*cI857 S_M1L/C51S _R::LacZα^+ ^J_WT _stf ^-^*)	[27]
SYP095	MC4100(λ*cI857 S_M1L/C51S _R::LacZα^+ ^J_WT _stf ^+^*)	[27]
SYP088	MC4100(λ*cI857 S_M1L/C51SS76C _R::LacZα^+ ^J_WT _stf ^-^*)	[27]
SYP096	MC4100(λ*cI857 S_M1L/C51SS76C _R::LacZα^+ ^J_WT _stf ^+^*)	[27]
RG26	MG1655(λ*cI857 Swt R::LacZα^+ ^J_245-2 _stf ^-^*)	This study
RG23	MG1655(λ*cI857 Swt R::LacZα^+ ^J_245-2 _stf ^+^*)	This study
RG31	MG1655(λ*cI857 Swt R::LacZα^+ ^J_1077-1 _stf ^-^*)	[17]
RG30	MG1655(λ*cI857 Swt R::LacZα^+ ^J_1077-1 _stf ^+^*)	This study
RG29	MG1655(λ*cI857 Swt R::LacZα^+ ^J_1127-1 _stf^-^*)	This study
RG32	MG1655(λ*cI857 Swt R::LacZα^+ ^J_1127-1 _stf^+^*)	This study

Phage strain

All phage strains used in this study were obtained by thermal induction of the above lysogens.

### Phage strain constructions

For phage λ, the host recognition and adsorption is mediated through interaction between the phage tail fiber J (encoded by gene *J*) and *E. coli *outer membrane protein LamB [55,56]. Side-tail fibers (Stf, encoded by the non-essential *stf *gene [54]) also contribute to host adsorption [27,54]. The lysis timing is determined by the activity of the S holin protein, encoded by the *S *gene [57,58]. The main goal of phage strain construction is to generate various isogenic λ strains that would differ in one or two of the following phenotypic traits: (*i*) the adsorption rate (via different *J *or *stf *alleles), (*ii*) the lysis time (via different *S *alleles), and (*iii*) the phage morphology (via the *stf *alleles). All these strains also carry the LacZα marker to facilitate image capture for plaque size measurement.

The method used in generating the λ strain carrying the *J_1077-1 _*allele [17] was adopted in this study to generate two more *J *alleles: *J_245-2 _*(carrying the T1040M mutation) and *J_1127-1 _*(carrying the Q1078R and L1127P mutations) [24]. Briefly, site-directed mutagenesis was used to introduce desired mutations into parental plasmids pZE1-J-stf and pZE1-J-stf+ [27]. The resulting plasmids were then transformed into SYP052 [27], a λ lysogen with the region between *J *and *orf401 *replaced by the *cam *marker. After thermal induction of the lysogen, only phage progeny that restored the tail fiber J function would be able to form plaques. Therefore, for each phage strain carrying the engineered *J *alleles, two associated states at the side tail fiber gene also existed: *stf^+ ^*or *stf^-^*. The primer sequences used for site-directed mutagenesis are shown in the Addition file 1.

To increase the contrast of the plaque against the background, we also introduced the *lacZα *gene into the λ genome by fusing it at the end of the endolysin *R *gene [27]. This is accomplished by transforming the plasmid pSwtRlacZblueRz [27], which carries the *R::lacZα *gene, into the lysogens containing the above constructed prophages. The desired recombinant λ strains were obtained by thermally inducing the lysogens and screening for blue plaques in the presence of IPTG and X-gal, and on the host strain XL1 Blue, which carries the complementary ω fragment of the *E. coli *β-galactosidase.

The identities of all strain constructs were confirmed by DNA sequencing.

### Construction of the *ΔompC::kan E. coli*

To construct an *E. coli *strain defective in OmpC production, we chose JW2203 from the Keio collection (CGSC#9781), which carries the desired *ΔompC768::kan *mutation [45], as our donor strain for P1 transduction. However, for some unknown reasons, we were unable to successfully P1-transduce the chromosomal region containing the *ΔompC768::kan *mutation into our XL1 Blue strain. To further our goal of determining the effect of phage morphology on plaque size, we constructed the strain IN731 by P1-transducing the mutation into the recipient strain SYP124, which is essentially the strain MG1655 but carrying the necessary ω-fragment expressed from *lcaZΔM15 *(unpublished data). Plaque size was determined by plating on SYP124 and its *ΔompC *counterpart, IN731.

### Standard PCR and DNA sequencing

Standard PCR reactions were performed using the following conditions: one cycle of 95°C for 1 min, followed by 30 cycles of 95°C for 30 s, 50°C for 30 s, and 72°C for several minutes, depending on the template size (using an extension of 1 min/Kb). PfuUltra (Stratagene, La Jolla, CA), a high-fidelity thermostable DNA polymerase, was used for amplification. The BigDye Terminator Cycle Sequencing kit (v3.1; ABI) was used for DNA sequencing according to the manufacturer's recommendation.

### Phage plating

To minimize variation, all plating conditions were standardized. A total of ~100 phages were mixed with fresh 100 μL of *E. coli *cells, prepared by two-fold dilution of overnight culture and grown at 37°C for 90 min in TB medium (5 g NaCl and 10 g Tryptone in 1 L H_2_O), and then incubated at room temperature for 20 min for pre-adsorption. In our experience, >90% of phages would be adsorbed onto the cells during the pre-adsorption period. The mixture was then mixed with 3 mL of molten H-top agar with IPTG and X-gal and overlaid on plates containing 40 mL LB-agar. Both the LB plates and the H-top agar were freshly prepared a few hours before use. The plates were then incubated for 18-22 h at 37°C before plaque size determination [17]. In our experience, the plaques would have reached their maximum size within this incubation period.

### Determination of phage adsorption rate

The protocol for adsorption rate determination, which is essentially the same as that used by Schlesinger [51], has been described previously [17]. Briefly, ~4.5 × 10^4 ^phages were mixed with 10 mL of *E. coli *XL1 Blue stationary phase cells (grown at 37°C for overnight in TB medium of 1% tryptone and 0.5% NaCl) in a flask with constant shaking (250 rpm/min) at 37°C. Two different cell concentrations-approximately 10^7 ^cells/mL for the Stf^+ ^phages and ~10^8 ^cells/mL for Stf^- ^the phages- were used because a lower cell concentration allows a more precise determination of free phage concentration for phages with a very high adsorption rate. At time 0, 5 and 15 min, 300 μl of the culture was withdrawn and immediately filtered on a 0.2 μm 96-well filter plate (Pall, East Hills, NY). The number of free phages in each sample was then determined by plating. Six replicates were performed for each phage strain. An exponential function of *y *= *be^-at ^*, where *a *and *b *are the parameters to be estimated, and *t *the time, was used to fit the data from individual experiments. The adsorption rate was obtained by dividing each of the estimated parameter *a *with its corresponding cell concentration. For more detail on how the adsorption rates were calculated, please see Additional file 3.

### Determination of plaque size

For each phage strain, images of four to five plates with phage plaques were taken with Qcount (Spiral Biotech, Inc.; Norwood, MA) and then analyzed using the ImageJ software (NIH). To convert the pixel count to surface area, we arbitrarily generated a computer printout with a known surface area and used it as the size standard. In this study, we found that 1 pixel = 0.01588 mm^2^.

Besides the phage traits, many other factors may also influence the plaque size. Several precautions were taken to minimize potential unintended effects. For example, to minimize plaque variation due to plating conditions [12], the plating conditions were standardized and only freshly prepared plates were used (see above). To reduce variation due to the timing of the formation of the initial attachments of phage particles, adequate amount of pre-adsorption time and high host concentrations (see above) were used to synchronize the timing of the formation of the initial infection centers before plating. This practice is especially critical for phages with low adsorption rates. To reduce the incidence of fusion of two nearby plaques, thus being measured as one large plaque, the number of phages on each plate was kept below 100. However, other factors, such as the edge effect (plaques on the edge of the plate were usually smaller), were unable to be controlled. Therefore, to further minimize potential skewing effects, plaque size distributions obtained from the four to five replicated plates were pooled, and the mode, rather than the mean, was used as the descriptive measure of these distributions. The determination of plaque size was performed nine times independently.

### Determination of plaque productivity

In order to estimate phage numbers in plaques (productivity), three random plaques from each of the four plates (used to estimate plaque size - see above) were obtained by taking agar plugs containing the plaques [17]. The 12 plaques were pooled together and then homogenized in 6 mL TB medium using a glass homogenizer with a Teflon plunger [17]. The homogenate was centrifuged for 10 min at 3000 × *g *(Eppendorf centrifuge 5702) at room temperature and the supernatant was then plated in triplicates at appropriate dilutions on a lawn of *E. coli *XL1 Blue. The experiment was performed nine times independently.

### Statistics

ANOVA and regressions (linear or quadratic) were used to detect significant relationships between phage traits and plaque properties. Lysis time (continuous) adsorption rate (continuous) and date (categorical) were used as explanatory variables in our statistical models. All statistical analyses were performed using the software package JMP, ver. 7.0.2 (SAS Institute Inc., Cary, NC) for the Macintosh computer. The 95% confidence intervals for various ratios shown in Figures 4A to 4F were calculated by following method devised by Fieller [59].

## Competing interests

The authors declare that they have no competing interests.

## Authors' contributions

The project was based on RG's original idea, supervised by INW, designed by RG and INW, λ strain constructions were carried out by RG, experiments were performed by RG and SK, statistical analyses performed by RG and INW, and the writing performed by RG, SK, and INW. All authors read and approved the final manuscript.

## Appendix

**Appendix:** List of models on plaque formation

Equation^1^	Main assumptions	Reference
(1)	phage propagating through a constant host density	[19], eqn. 18

(2)	phage adsorption/desorption processes are fast relative to cell death rate	[20], eqn. 6a

(3)	larger burst size	[20], eqn. 6b

(4)	phage adsorption/desorption processes are slow relative to cell death rate	[20], eqn. A8

(5)	phage adsorption process is fast relative to cell death rate	[20], eqn. A9

(6)	hindered diffusion through a high constant host density	[23], eqn. 14, solution 1

(7)	hindered diffusion through a high constant host density	[23], eqn. 14, solution 2

^1 ^The variables are: *c*, the plaque wavefront velocity; *D*, the virion diffusivity; *N_o_*, the lawn bacterial density; *L*, the latent period (or lysis time); *k_1_*, the adsorption constant of the phage particle; *k_-1_*, the desorption constant; and *k_2_*, the rate constant for lysis.

## Supplementary Material

Additional file 1 **Model testing**. Testing of models on plaque size and plaque productivity. Click here for file

Additional file 2 **Primer sequences and plasmids**. PCR primer sequences and plasmids used to generate isogenic λ strains. Click here for file

Additional file 3 **Examples of adsorption rate data and adsorption curves**. Examples of adsorption rate data and adsorption curves for the highest (J_1077 _Stf^+^) and lowest (J_WT _Stf^-^) adsorption rate phages used in this study. Click here for file
