# Supplementary material for: Effects of bacteriophage traits on plaque formation
Source: BMC Microbiol. 2011 Aug 9;11:181. doi: 10.1186/1471-2180-11-181 (PMC3176204; doi:10.1186/1471-2180-11-181)
Supplement: Additional file 1 — Model testing. Testing of models on plaque size and plaque productivity. [file 1471-2180-11-181-S1.DOC]

**Additional file 1**

**Model testing**

Abedon and Culler [16, 22] have conducted the most comprehensive review to date on models of phage plaque enlargement.

Since we did not measure all quantities directly, we cannot test the model predictions directly. However, we can test the validity of these models by comparing the ratio of plaque sizes of any given two phage strains. This is made possible by the fact that all of our phage strains are isogenic and, therefore, the plaque size or plaque productivity difference between any two strains would simply be due to the difference in phage traits we specifically changed.

To take advantage of our collection of isogenic phage strains and identical experimental condition, the following assumptions were made to facilitate the comparison:

(1) $\frac{c_{1}}{c_{2}}=\frac{r_{1}}{r_{2}}=\frac{\sqrt{A_{1}}}{\sqrt{A_{2}}}$, where *r* is the radius and *A* the area of the plaque. This is because all plating were incubated for the same duration, thus rate of plaque growth can be converted to size of growth.

(2) Phages having the same morphology would have the same diffusivity. In our study, we have two different phage morphology: Stf^+^ and Stf^-^.

(3) The burst size *B* can be expressed as *B* = *m*(*L* -*E*), where *m* is the virion assembly rate and *E* the eclipse period. This linear relationship should hold well for the range of *L* we have in our phage collection [26]. Also, in this study, all phage strains should have the same *m* and *E*, which has been previously determined to be ~7.7 phages/min and ~28 min, respectively [26]. In all our cases, *B* >> 1, therefore, *B* - 1 ≈ *B*.

(4) The empirically determined *N_0_* is ~8.5 × 10^8^ cells/mL of top agar gel after overnight incubation (data not shown).

(5) The empirically determined adsorption rate *a* can be expressed as *k_1_*/*k_-1_*. This is because *a* is the net result of adsorption-desorption of the virus particles. However, for the equations with only the *k_1_* term, then we would substitute *a* as *k_1_*, thus effectively making the assumption that the phage adsorption rate is mainly determined by its adsorption kinetics and the desorption rate can be ignored.

(6) Since *k_2_* is the rate of converting the phage-bacterium complex into lysis, therefore, $k_{2}\propto\frac{1}{L}$.

**I. Testing models on plaque size**

Based on the above assumptions, the predicted plaque radius ratios are listed below:

| Ratios of plaque size based on model prediction as summarized by Abedon and Culler [22]. | | |
| --- | --- | --- |
| Equation | Different adsorption rate | Different lysis time |
| (1) | n/a | $\frac{r_{1}}{r_{2}}=\frac{\sqrt{L_{2}}}{\sqrt{L_{1}}}$ |
| (2) | $\frac{r_{1}}{r_{2}}=\frac{\sqrt{a_{1}}}{\sqrt{a_{2}}}(\frac{1+N_{0}a_{2}}{1+N_{0}a_{1}})$ | $\frac{r_{1}}{r_{2}}=\frac{\sqrt{L_{2}}}{\sqrt{L_{1}}}(\frac{\sqrt{L_{1}-E}}{\sqrt{L_{2}-E}})$ |
| (3) |  |  |
| (4) | $\frac{r_{1}}{r_{2}}=\frac{\sqrt{a_{1}}}{\sqrt{a_{2}}}$ | $\frac{r_{1}}{r_{2}}=\frac{\sqrt{L_{1}-E}}{\sqrt{L_{2}-E}}$ |
| (5) | $\frac{r_{1}}{r_{2}}=\frac{\sqrt{a_{2}}}{\sqrt{a_{1}}}$ | $\frac{r_{1}}{r_{2}}=\frac{L_{2}}{L_{1}}(\frac{L_{1}-E}{L_{2}-E})$ |
| (6) | $\frac{r_{1}}{r_{2}}=\frac{\sqrt{a_{1}}}{\sqrt{a_{2}}}(\frac{\sqrt{1+LBN_{0}a_{2}}}{\sqrt{1+LBN_{0}a}_{1}})$ | $\frac{r_{1}}{r_{2}}=\frac{\sqrt{L_{1}-E}}{\sqrt{L_{2}-E}}(\frac{\sqrt{1+L_{2}B_{2}N_{0}a}}{\sqrt{1+L_{1}B_{1}N_{0}a}})$ |
| (7) | na | $\frac{r_{1}}{r_{2}}=\frac{\sqrt{L_{2}}}{\sqrt{L_{1}}}$ |

For example, the expected ratio of plaque radius between λ Stf^+^ J_1127-1_ and wt λStf^+^ J_wt_, as predicted by eqn. 6, would be $\frac{r_{J1127-1}}{r_{Jwt}}=\frac{\sqrt{1.18\times10^{-8}}}{\sqrt{1.03\times10^{-8}}}(\frac{\sqrt{1+51.0\times170\times8.5\times10^{8}\times1.03\times10^{-8}}}{1+51.0\times170\times8.5\times10^{8}\times1.18\times10^{-8}}=1.0$, where 1.18 × 10^-8^ and 1.03 × 10^-8^ are estimated adsorption rates for λ Stf^+^ J_1127-1_ and wt λStf^+^ J_wt_, respectively, 51 is the lysis time for wt S holin, and 170 is the burst size, both values are from Wang [26].

**II. Testing models on plaque productivity**

The most comprehensive treatment on plaque productivity is also found in Abedon and Culler [22]. The authors took advantage of the fact that models of plaque enlargement [16] can be used to model the productivity (fecundity, in their terminology) of the plaque. It is assumed that all the bacterial cells within the plaque volume, be it a fully formed sphere, a truncated cylinder, or any shape in between, would be infected and turned into phage progeny with a constant conversion rate of the phage burst size. That is, the ratio of phage productivity *p* can be expressed simply as

$\frac{p_{1}}{p_{2}}=\frac{B_{1}}{B_{2}}(\frac{r_{1}}{r_{2}})^{3}$, where *B* is the burst size and *r* the plaque radius.

Using Table A.2, we can also test models on plaque productivity. The above equation can be used in the case in which all isogenic strains have different lysis times.

For the case of comparing between isogenic strains that differed in adsorption rate, the above equation can be simplified as

$\frac{p_{1}}{p_{2}}=(\frac{r_{1}}{r_{2}})^{3}$, because all strains have the same lysis time and , thus, burst size.

However, in the case of comparing between isogenic strains that differed in lysis times, the ratio of $\frac{p_{1}}{p_{2}}=\frac{B_{1}}{B_{2}}(\frac{r_{1}}{r_{2}})^{3}$ are used. Previously determined burst sizes for each lysis time variant [26] are used.
