# Supplementary material for: Effects of bacteriophage traits on plaque formation
Source: BMC Microbiol. 2011 Aug 9;11:181. doi: 10.1186/1471-2180-11-181 (PMC3176204; doi:10.1186/1471-2180-11-181)
Supplement: Additional file 2 — Primer sequences and plasmids. PCR primer sequences and plasmids used to generate isogenic λ strains. [file 1471-2180-11-181-S2.DOC]

**Additional file 2**

**Primer sequences and plasmids**

| Primer | Sequence (5' to 3') | References |
| --- | --- | --- |
| Charbit148for | GATTAAGAAACAGGCGCCGGGCATCAGCGTGG | This study |
| Charbit148rev | CCACGCTGATGCCCGGCGCCTGTTTCTTAATC | This study |
| Charbit245-1for | GGTGCTTCCGCTGATGTTTCGCGGAAGTAAG | This study |
| Charbit245-1rev | CTTACTTCCGCGAAACATCAGCGGAAGCACC | This study |
| Charbit247-6for | GGCGAACGAGGCGGCACAGGTGTTCTCCCG | This study |
| Charbit247-6rev | CGGGAGAACACCTGTGCCGCCTCGTTCGCC | This study |
| Charbit1077-1afor | GATGGCGCGGCGAACGTGGCGGTACAGGTGTTC | [17] |
| Charbit1077-1arev | GAACACCTGTACCGCCACGTTCGCCGCGCCATC | [17] |
| Charbit1077-1bfor | GGCGCGGCGAACGAGTCGGTACAGGTGTTCTC | [17] |
| Charbit1077-1brev | GAGAACACCTGTACCGACTCGTTCGCCGCGCC | [17] |
| Charbit1127-1for | GAACGAGGCGGTACGGGTGTTCTCCCGTATTG | This study |
| Charbit1127-1rev | CAATACGGGAGAACACCCGTACCGCCTCGTTC | This study |
|  | | |
| Plasmids | Description | References |
| pZE1-J-stf | Contains the genomic region of λ, encompassing part of *J*, entire *lom*, *orf401*, and part of *orf314* (*orf401* and *orf314* are the result of a frameshift mutation by a single cytocine (C) deletion at the *stf* gene) | [27] |
| pZE1-J-stf+ | The same as the pZE1-J-stf, except that a single C was inserted to restore the functional *stf* gene | [27] |
