# Supplementary material for: Effects of bacteriophage traits on plaque formation
Source: BMC Microbiol. 2011 Aug 9;11:181. doi: 10.1186/1471-2180-11-181 (PMC3176204; doi:10.1186/1471-2180-11-181)
Supplement: Additional file 3 — Examples of adsorption rate data and adsorption curves. Examples of adsorption rate data and adsorption curves for the highest (J1077 Stf+) and lowest (JWT Stf-) adsorption rate phages used in this study. [file 1471-2180-11-181-S3.DOC]

**Additional file 3**

**Examples of adsorption rate data and adsorption curves**

**(A) Adsorption rate data**

| **Jwt Stf-** | **Replicate** | | | | | |
| --- | --- | --- | --- | --- | --- | --- |
| **Time (min)** | **1** | **2** | **3** | **4** | **5** | **6** |
| **0** | 2.99E+04 | 6.34E+04 | 5.44E+04 | 3.72E+04 | 4.57E+04 | 4.54E+04 |
| **5** | 2.91E+04 | 6.40E+04 | 4.73E+04 | 3.62E+04 | 3.81E+04 | 4.41E+04 |
| **15** | 2.89E+04 | 5.62E+04 | 4.25E+04 | 3.65E+04 | 3.68E+04 | 4.35E+04 |
| **Estimated slope** | -1.80E-03 | -8.70E-03 | -1.56E-02 | -1.00E-03 | -1.29E-02 | -2.60E-03 |
| **Average cell concentration** | 6.25E+07 | 3.01E+08 | 9.00E+07 | 7.50E+07 | 8.75E+07 | 4.78E+07 |
| **Calculated adsorption rate** | -2.88E-11 | -2.89E-11 | -1.73E-10 | -1.33E-11 | -1.47E-10 | -5.45E-11 |
| **J1077 Stf+** | **Replicate** | | | | | |
| **Time (min)** | **1** | **2** | **3** | **4** | **5** | **6** |
| **0** | 3.20E+04 | 5.01E+04 | 5.14E+04 | 4.69E+04 | 4.36E+04 | 4.53E+04 |
| **5** | 1.98E+04 | 2.63E+04 | 3.02E+04 | 3.07E+04 | 2.70E+04 | 2.88E+04 |
| **15** | 9.70E+03 | 1.26E+04 | 1.18E+04 | 1.07E+04 | 9.00E+03 | 1.14E+04 |
| **Estimated slope** | -7.84E-02 | -8.94E-02 | -9.75E-02 | -9.95E-02 | -1.06E-01 | -9.21E-02 |
| **Average cell concentration** | 9.00E+06 | 8.75E+06 | 7.13E+06 | 7.73E+06 | 5.63E+06 | 4.75E+06 |
| **Calculated adsorption rate** | -8.71E-09 | -1.02E-08 | -1.37E-08 | -1.29E-08 | -1.88E-08 | -1.94E-08 |

**(B) Examples of adsorption curves**
